# Small airway disease as a key factor in COPD: new perspectives and insights

**DOI:** 10.3389/fmed.2025.1648612

**Published:** 2025-09-26

**Authors:** Ruoyi Zhou, Haojie Wang, Yulu Zhang, Jieming Mai, Liuliu Yang

**Affiliations:** The First Affiliated Hospital of Guangzhou University of Chinese Medicine, Guangzhou, China

**Keywords:** COPD, small airways, lung mechanics, early diagnosis of COPD, small airway disease

## Abstract

Small airways–defined as bronchioles <2 mm in internal diameter that lack cartilaginous support–are frequently involved in the earliest stages of chronic obstructive pulmonary disease (COPD). While COPD is defined per GOLD by persistent post-bronchodilator airflow limitation, small-airway dysfunction can precede spirometric abnormality, motivating earlier, imaging- and physiology-based detection (Agustí et al., 2023). Pathological progression typically begins with loss and stenosis of terminal bronchioles, followed by mucus retention/plugging, fibrotic remodeling, chronic inflammation, microvascular abnormalities, and cellular senescence, ultimately resulting in irreversible impairment of gas exchange. Early diagnosis remains difficult, but a suite of advanced non-invasive modalities–including impulse oscillometry system/forced oscillation techniques (IOS/FOT), single- and multiple-breath washout tests, high-resolution CT with parametric response mapping (PRM), nuclear medicine approaches (e.g., SPECT), dynamic measurements of lung compliance, and Fluorine-19 (^19^F) MRI–combined with artificial intelligence markedly improve the sensitivity and specificity for detecting small-airway disease. Therapeutic strategies that target cellular senescence and fibrotic pathways–such as senolytics and antifibrotic interventions–are showing promise, particularly approaches that clear senescent cells or block pro-fibrotic signaling. The integration of single-cell omics, high-resolution microvascular imaging, and molecularly targeted therapies is expected to accelerate precision diagnostics and enable personalized early interventions. This review summarizes recent insights into small-airway physiology, key pathophysiological and molecular mechanisms, and current pharmacological strategies, and emphasizes the clinical principle of “early detection, early diagnosis, early intervention” for managing COPD-related small-airway disease.

## Background

Small airway disease (SAD) plays a central role in the pathogenesis of chronic obstructive pulmonary disease (COPD), a role that has only recently been fully recognized (2). Small airways, defined as small bronchi with an internal diameter less than 2 mm and no cartilage support, comprise the majority of the distal airway cross-sectional area. Due to their narrow lumen and lack of cartilage support, these airways are extremely sensitive to inhaled noxious particles and irritants, yet are clinically invisible, earning them the nickname “silent zones (3).” Early functional impairment of the small airways often precedes the onset of clinical symptoms, abnormal lung function, or radiographic changes.

Historically, SAD was considered a secondary manifestation of established COPD. However, pathological and high-resolution imaging evidence has changed this understanding: progressive destruction and loss of terminal bronchioles can precede alveolar parenchymal destruction or a significant decline in overall lung function, suggesting that SAD may be the initial step in the disease cascade (4, 5). A multimodal analysis (6) found significant differences in CT small airway/gas trapping indices (including PRM-calibrated fSAD), clinical symptoms, and blood transcriptomic profiles between smokers with COPD, asthma, and ACO. This study linked imaging-defined SAD to molecular phenotypes, supporting the use of SAD as a basis for phenotyping airway diseases and stratifying diagnosis and treatment.

This paradigm shift also explains why patients with similar traditional lung function measures exhibit significant differences in the burden of airway structural damage and clinical outcomes. Research has revealed that SAD reflects the convergence of multiple pathological processes, including chronic inflammation, mucus hypersecretion, epithelial-mesenchymal transition (EMT) and fibrotic remodeling, microvascular rarefaction, epithelial barrier dysfunction, and cellular senescence. Signaling pathways such as TGF-β, PI3K/Akt, and NF-κB play key roles in these processes, collectively regulating inflammation, mucus secretion, EMT, fibrosis, and cellular senescence, driving progressive airway narrowing and ultimately impairing gas exchange. These theoretical and mechanistic advances have revealed several key clinical questions. While conventional pulmonary function testing remains the cornerstone of diagnosing airflow limitation, its sensitivity for early detection of SAD is insufficient (7). Emerging physiological and imaging tools–such as impulse oscillometry system single- and multiple-breath washout tests, high-resolution CT (HRCT) combined with parametric response mapping (PRM), molecular magnetic resonance imaging, and targeted nuclear medicine imaging–when combined with artificial intelligence applications, have the potential to enhance detection capabilities. However, further validation, standardization, and integration into clinical workflows are required. In terms of treatment, anti-aging drugs, anti-fibrosis drugs, and targeted interventions for the above-mentioned signaling pathways have shown potential in preclinical studies, but their translational applications still face challenges such as target specificity, intervention timing, and patient stratification.

## Physiological characteristics of the small airway

Small airways are defined as those with a diameter of less than 2 mm and are considered part of the distal airway system, comprising membranous bronchioles, respiratory bronchioles, and alveolar ducts (8). The structures typically originate from the 8th to 15th generations of branches and terminate in the terminal bronchioles. They may range several centimeters in length and exhibit an irregular oval cross-section. The tracheal wall is thin, and contains smooth muscle and a minimal amount of cartilage (9). The inner wall is lined by ciliated columnar epithelial cells. The more distal respiratory bronchioles transition from columnar to cuboidal epithelium and subsequently lead into the alveolar ducts and cavities, which are lined with flat epithelium (10). Additionally, dendritic cells and macrophages residing in the small airways identify and eliminate pathogens, thereby forming an immune barrier. In contrast to the large airways, gas flow in large airways is predominantly turbulent, whereas small airways exhibit predominantly laminar flow, characterized by a lower Reynolds coefficient. Therefore, alterations in gas density have minimal or negligible effects on the resistance of small airways, whereas gas density significantly influences the resistance of large airways (9). Another physiological distinction between the small and large airways is that the liquid lining of the small airways possesses surfactant-like properties, particularly during exhalation. This low surface tension prevents the small airways from collapsing as lung volume decreases (11). These findings suggest that active substances also play a role in regulating gas diffusion distance and lubricating ciliary movement, thereby helping to maintain airway patency. At the end of inspiration, small airways are susceptible to collapse due to a decreased pulling force, leading to dynamic alveolar closure, increased dead space, and reduced gas exchange efficiency. Under normal circumstances, small airways contribute approximately 10% of total airway resistance. However, in the early stages of COPD, small airway resistance significantly increases, reflecting small airway dysfunction (9).

## Initiation phase

### Epithelial damage–EMT (epithelial-mesenchymal transition)—impaired barrier function–mucus obstruction

The small airway epithelium is the main defense barrier of the respiratory tract against inhaled harmful particles and pathogens. However, prolonged exposure to harmful substances in cigarette smoke and air pollution leads to a cyclical process of repeated damage and continuous repair of the small airway epithelium. The main features of initial epithelial injury are mucociliary dysfunction, goblet cell hyperplasia, and disruption of tight junction proteins, changes that together impair mucociliary clearance and compromise barrier integrity (12). This pathologic progression not only enhances airway hypersensitivity to exogenous stimuli, but also sets the stage for a persistent inflammatory response. In a chronic inflammatory microenvironment, damaged epithelial cells can initiate EMT by activating multiple signaling pathways, such as transforming growth factor-β (TGF-β), Wnt/β-catenin, and the Notch pathway (13). EMT is a process of cellular phenotypic plasticity in which epithelial cells gradually lose polarity and intercellular connections and acquire a mesenchymal-like phenotype (including enhanced migration ability and increased collagen deposition), thereby driving early fibrosis and airway remodeling (14). At the same time, STAT3 activation can amplify pro-fibrotic and pro-inflammatory signals, while dysregulation of the PINK1-Parkin pathway, responsible for mitochondrial quality control, promotes epithelial damage and the progression of EMT by increasing mitochondrial dysfunction and reactive oxygen species (ROS) production. The above signaling pathways synergistically promote epithelial-mesenchymal transition in this stage, laying the molecular foundation for subsequent pathological changes (15). The progression of EMT with fibrotic processes leads to further deterioration of small airway barrier function. Firstly, basement membrane thickening and collagen deposition increase airway stiffness and decrease gas exchange efficiency. Secondly, a compromised barrier promotes infiltration of inflammatory cells and noxious particles into the deeper layers of the airway, thus creating a self-perpetuating cycle of injury and inflammation (16). Although molecular features of EMT (such as downregulation of E-cadherin and upregulation of vimentin) are commonly observed in the small airways of smokers, their exact contribution to airway obstruction remains controversial (17). At the same time, epithelial damage with impaired barrier function promotes the secretion of abnormal mucus in small airways. In patients with COPD, the incidence of small airway mucus plugging is elevated and positively correlates with disease severity (18). Triggered by initial epithelial injury, goblet cell proliferation, mucociliary dysfunction, and up-regulation of mucin (e.g., MUC5AC and MUC5B) expression combine to further impair mucociliary clearance (19–23). Cigarette smoke and air pollution directly induce goblet cell proliferation and promote mucin secretion, which in turn leads to mucus embolism. Simultaneously, secretory immunoglobulin A (SlgA) levels on the surface of small airways are reduced and polymeric immunoglobulin receptor (plgR) expression is decreased. Together, these changes compromise the epithelial antimicrobial barrier and increase susceptibility to infection (24–27). Mucus retention not only impedes airflow on a physical level, but also provides a suitable environment for pathogens to proliferate. This can exacerbate localized inflammation, which in turn creates a self-perpetuating cycle of pathology. This is the initial and critical stage in the progression from small airway disease (SAD) to COPD (5). Therefore, the pathological events from small airway epithelial injury to EMT, barrier damage, and mucus plugging are intertwined, forming a continuous pathological chain: epithelial injury–EMT/TGF-β/STAT3/PINK1-Parkin–barrier disruption–mucus plugging. This is a chain reaction that reveals important molecular mechanisms of early COPD lesions. Future interventions targeting the EMT process (such as TGF-β inhibitors or drugs that regulate oxidative stress) may provide new ideas for the early prevention and treatment of COPD (28).

## Expansion Phase

### Immune cell recruitment – mediator release – critical pathway activation— transition to senescence

**FIGURE 1:**
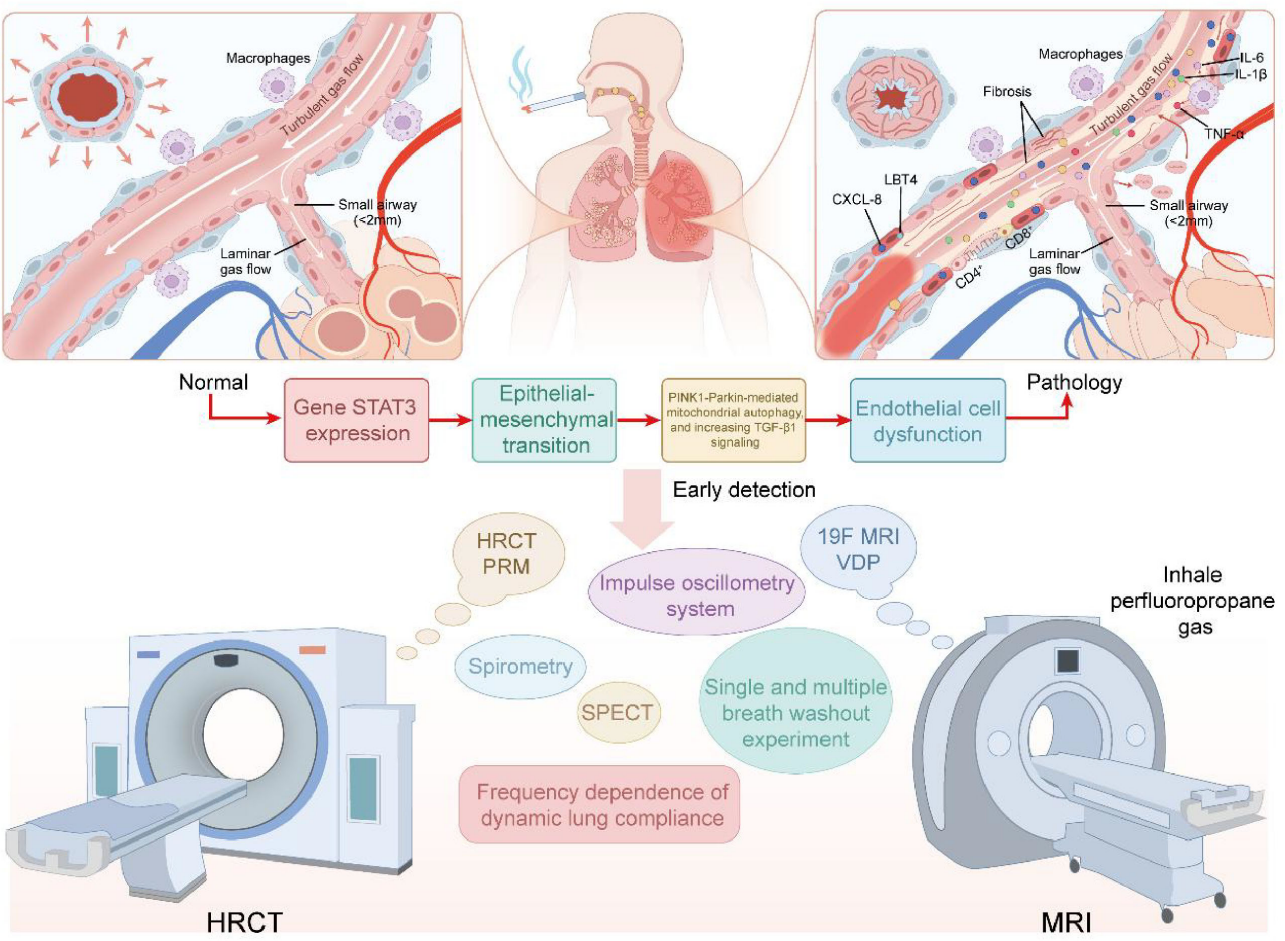
Top panels show enlarged small-airway cross-sections (left: near-normal; right: pathological changes including mucus plugging, epithelial injury, inflammatory-cell infiltration, and focal fibrosis). The middle panel depicts a molecular–cellular cascade–exposure (e.g., cigarette smoke or air pollution) → STAT3/TGF-β-driven epithelial-to-mesenchymal transition (EMT), PINK1–Parkin-related mitochondrial/autophagy dysfunction → endothelial dysfunction–illustrating the principal sequence *Initiation → Expansion → Remodeling*. The lower panel lists imaging and physiological modalities for early detection and phenotyping (HRCT-PRM, CT arterial pruning, 19F-MRI ventilation defect percentage (VDP), impulse oscillometry system (IOS), multiple-breath washout [MBW], SPECT, and conventional spirometry), emphasizing the complementary roles of imaging and functional assays in identifying individuals with functional small-airways disease (fSAD) and gas trapping despite preserved spirometry (pre-COPD/early SAD). This diagram is conceptual; several mechanistic links remain to be confirmed by longitudinal and mechanistic studies.

Small airway inflammation is induced by chronic exposure to cigarette smoke and atmospheric particulate matter. These stimuli activate airway epithelial cells and alveolar macrophages, leading to the release of pro-inflammatory mediators (e.g., TNF-α, IL-1β, IL-6) and chemokines (e.g., CXCL8, LTB4) (Figure 1), which recruit neutrophils into the airway wall, ultimately driving the inflammatory response in small airways (29). In the small airways of COPD, neutrophils represent the predominant and earliest infiltrating cells responding to cigarette smoke, followed sequentially by the recruitment of macrophages and CD4^+^/CD8^+^ T lymphocytes. These immune cells exacerbate tissue injury through cytotoxicity and the release of inflammatory mediators, and are closely associated with the development of emphysema (30–35). For instance, cigarette smoke and particulate matter can amplify and perpetuate a chronic inflammatory milieu by activating the NF-κB and p38 MAPK signaling pathways, thereby driving the sustained secretion of cytokines such as TNF-α, IL-1β, IL-6, and IL-8. Currently, oxidative stress can activate the PI3K/Akt/mTOR pathway, whose upregulation in the airways of elderly COPD patients has been experimentally demonstrated. This pathway further exacerbates the inflammatory response by suppressing SIRT1/6 activity and enhancing NF-κB pathway activity (31). Approximately 20%–40% of patients with COPD exhibit an inflammatory phenotype characterized by eosinophilic infiltration. This phenotype correlates strongly with small airway dysfunction and often predicts a favorable response to inhaled corticosteroid (ICS) therapy (36, 37). Chronic inflammation also promotes goblet cell hyperplasia and hypertrophy of mucous glands, thereby elevating both the volume and viscosity of secreted mucus. The consequent formation of mucus plugs contributes to the worsening of airflow obstruction (29, 38). Notably, persistent inflammation and oxidative stress can drive DNA damage, telomere shortening, and the accumulation of reactive oxygen species (ROS), thereby inducing cells to enter a state of senescence (39).

## Cellular senescence, the senescence-associated secretory phenotype (SASP), miR-34a, and sirtuins (SIRT1/6)

Chronic inflammation and oxidative stress induce epithelial cells, fibroblasts, and endothelial cells into a senescent phenotype through the ATM/ATR-p53/p21 pathway (39). Senescent cells exhibit permanent growth arrest and secrete a group of pro-inflammatory and pro-fibrotic factors, the senescence-associated secretory phenotype (SASP), which mainly includes IL-6, IL-8, MMP-9, MMP-12, etc. These factors can amplify inflammation, promote ECM remodeling and induce functional decline of adjacent cells in a paracrine manner (40, 41). Meanwhile, microRNA-34a (miR-34a) has been reported to be upregulated in the regulation of aging and oxidative stress, and promotes the formation of aging phenotypes and the persistence of SASP by downregulating SIRT1/6 (key deacetylases involved in antioxidant and repair responses) (42–44). Within fibroblasts, SASP factors stimulate the accumulation of collagen and laminin, resulting in subepithelial fibrosis and consequent stiffening of the airway wall. Additionally, dysregulated iron metabolism amplifies ROS production through the Fenton reaction, which in turn drives both fibroblast senescence and extracellular matrix (ECM) remodeling (45). Short peptides produced after ECM is cleaved by proteases can act as damage-associated molecular patterns (DAMPs) or direct chemokines, activating receptors such as TLRs, inducing cytokine release and promoting neutrophil recruitment. Studies in the lungs have shown that these short peptides can prevent immune cell infiltration and affect the process of repairing fibrosis in chronic severe lung cancer (46). Furthermore, emerging evidence indicates that substance P, a neuropeptide, may mediate neurogenic inflammation in response to epithelial irritation and damage caused by cigarette smoke. This neurogenic response exacerbates mucus hypersecretion and inflammatory cell infiltration in the small airways (47). Moreover, studies have indicated a potential role for substance P in mediating repair mechanisms after epithelial damage in the small airways. Nevertheless, aberrant overexpression of this neuropeptide may promote the development of fibrosis (48). Treatment with a neurokinin 1 (NK1) receptor antagonist attenuates airway inflammation and bronchial hyperresponsiveness, leading to concomitant improvement in lung function (49). A study found that the migration of airway smooth muscle cells in asthma model rats was significantly increased. The NK1 receptor antagonist WIN62577 can inhibit this migration and reduce the expression of α-tubulin, suggesting that it can alleviate airway remodeling (50). Another study showed that in a bleomycin-induced rat pulmonary fibrosis model, the NK1R antagonist aprepitant significantly reduced the activity of the TGF-β/Smad3 signaling pathway and the expression of inflammatory cytokines (such as TNF-α and IFN-α), while increasing the level of antioxidants, exerting anti-fibrotic and anti-inflammatory effects (51). These results suggest that NK1R blockade may alleviate disease progression by reducing airway inflammation, fibrosis, and remodeling. However, the current relevant mechanism research and clinical evidence are still in the preliminary stage.

In summary, the inflammation-senescence axis drives the progression of small airway disease toward COPD, forming a vicious cycle. In this cycle, chronic inflammation promotes the release of pro-inflammatory mediators and chemokines, activates key signaling pathways, and elevates ROS production, collectively culminating in cellular senescence. Senescent cells release the senescence-associated secretory phenotype (SASP), which exacerbates inflammation, promotes extracellular matrix (ECM) remodeling, and induces secondary senescence in neighboring cells. This process establishes a self-perpetuating cycle that serves as a persistent mechanism underlying small airway structural damage and progressive airflow limitation, thereby driving disease worsening. This process thereby establishes the pathological foundation for subsequent airway structural remodeling. Emerging evidence suggests that the NLRP3 inflammasome acts as a pivotal amplifier of inflammation and is strongly implicated in the acute exacerbations of chronic respiratory diseases. Upon stimulation by reactive oxygen species (ROS), extracellular ATP, or lysosomal destabilization, NLRP3 activation drives the maturation and release of pro-inflammatory cytokines such as IL-1β and IL-18, which in turn promote neutrophil recruitment and tissue injury. This feed-forward mechanism establishes a ROS–IL-1β amplification loop, thereby perpetuating airway inflammation and contributing to recurrent exacerbations. Notably, experimental data indicate that pharmacological or genetic inhibition of NLRP3 can effectively interrupt this cycle, dampening the inflammatory cascade within the small airways. Furthermore, a combinatorial approach targeting both NLRP3 activation and oxidative stress has been proposed as a promising therapeutic strategy, with the potential to restore small airway function, mitigate structural injury, and ultimately prevent disease progression (52).

## Remodeling phase

### Proliferation of smooth muscle cells–Fibrosis–Vascular Pruning

Airway smooth muscle (ASM) in COPD small airway pathology commonly exhibits cellular hyperplasia and hypertrophy, leading to airway wall thickening, increased airway contractility, and directly contributing to small airway stenosis and airflow limitation (53). Epithelial and immune cells release various pro-proliferative/pro-fibrotic factors (such as TGF-β and PDGF) when epithelial injury and chronic inflammatory microenvironment occur. These factors stimulate ASM proliferation and induce phenotypic transformation by acting through the PI3K/Akt/mTOR and MAPK signaling pathways. Additionally, tobacco-induced Oxidative stress is able to amplify these pro-proliferative signals and further promote ASM hyperplasia (53, 54). Concomitant with ASM hyperplasia, local epithelium may undergo epithelial-mesenchymal transition (EMT). Meanwhile, recruited or activated fibroblasts differentiate into α-SMA-positive myofibroblasts, which synthesize large amounts of type I/III collagen and laminin, resulting in excessive sub-basement membrane ECM deposition and solidification of the airway wall, thereby reducing lung compliance and exacerbating irreversible stenosis (5, 54). In this process, the imbalance between Matrix Metalloproteinases (MMPs) and Tissue Inhibitors of Metalloproteinases (TIMPs) shows a “bidirectional effect”. On one side, ECM degradation mediated by MMP leads to elastic fiber rupture and emphysematous changes. On the other side, ECM fragments and pro-fibrotic signals trigger fibroblast activation and collagen deposition, ultimately resulting in spatially heterogeneous remodeling characterized by the coexistence of destruction and deposition (54, 55). The molecular mechanisms of how STAT3 activation and PINK1-Parkin regulation of mitochondria bridge epithelial damage and fibrosis have been described in detail in the initial section and will not be repeated here. From a biomechanical perspective, ASM hyperplasia and ECM deposition alter local tissue tension and generate mechanical compression, promoting distal capillary dysfunction and structural loss (capillary rarefaction). Concurrently, chronic inflammation, endothelial injury, and intermittent hypoxia also drive arteriolar intima-media thickening (arterial remodeling). These two types of vascular changes (arterial remodeling and capillary rarefaction) negatively affect clinical outcome (FEV1, DLCO, etc.) by increasing pulmonary vascular resistance or decreasing gas diffusion capacity, respectively (56, 57). Further evidence from the COPDGene cohort demonstrated that CT-based showed that “Arterial pruning”–decrease in distal small arterial vessel volume–was associated with emphysema progression, pulmonary function deterioration, and adverse outcomes, suggesting that vascular pruning is not only a concomitant change but also serves as an independent driving factor of disease progression (56, 58). Longitudinal study evidence from the COPDGene cohort indicates that, after excluding the influence of baseline lung parenchymal destruction, baseline arterial pruning can predict accelerated emphysema progression and decline in lung function (56); And in smokers without obvious COPD, the early presence of arterial pruning suggests its pathogenic role in increasing pulmonary vascular resistance and promoting right ventricular remodeling (58). Although the pruning process is regulated by the inflammatory reaction (e.g., cigarette smoke-induced endothelial-mesenchymal transition) (59, 60), and may be exacerbated by fibrosis compression (61), its independent impact on the disease course and clinical outcome highlights its core importance in the pathogenesis. Arterial remodeling and capillary rarefaction are two distinct angiopathies, each having unique physiological impacts. Arterial remodeling is manifested by structural alterations in muscular arteries, including intimal thickening and reduction in lumen diameter. These changes can increase pulmonary vascular resistance, induce pulmonary hypertension, lead to right ventricular dysfunction, and consequently cause hemodynamic impairment and limited motor capabilities (62, 63). In contrast, capillary rarefaction refers to decrease in capillary density. Gas exchange is directly impaired by reducing diffusing capacity (DLCO) and disrupting ventilation-perfusion matching, ultimately resulting in hypoxemia and ventilation/perfusion ratio (V/Q) imbalance (64–66). It is important that there is a synergistic effect between these two lesions: hypertension secondary to arterial remodeling exacerbates capillary loss, while capillary rarefaction further increases vascular resistance, collectively contributing to clinical deterioration in COPD patients.

In summary, ASM proliferation, ECM fibrosis, and vascular pruning are spatiotemporally coupled. molecular signals driven by epithelium/immune cells (TGF-β, STAT3, ROS, miRNA, etc.) not only directly promote the functional activation of smooth muscle and fibroblasts that leads to fibrosis, but also exacerbate tissue hypoxia and progressive injury by altering tissue mechanics and microvascular blood supply. Therefore, multi-target comprehensive treatment for this axis holds significant translational potential and merits further exploration in clinical trials (15, 55, 56).

## Single-cell omics unveils molecular lineages and remodeling mechanisms of the small airways

In small airway stenosis, fibroblast-driven ECM remodeling is the core of structural stenosis, while persistent inflammation dominated by neutrophils and macrophages provides the cellular and enzymatic basis for this process (such as MMPs and ROS), promoting the degradation and redeposition of ECM (67, 68). Recent single-cell and multimodal omics studies have revealed, at both molecular and cellular levels, how distinct cell populations aggregate early and orchestrate extracellular matrix (ECM) remodeling. Single-cell atlases of terminal bronchioles demonstrate that, in the early stages of COPD (GOLD I–II), M1-like macrophages and neutrophils preferentially accumulate at alveolar attachment sites, where their presence correlates with elastic fiber degradation. These findings indicate that immune cell infiltration and ECM injury occur in close temporal and spatial proximity, synergistically driving the initiation of tissue destruction (69). Multiple single-cell studies, including global scRNA-seq analyses of COPD alveolar regions and airway terminals, have uncovered a complex network of ligand–receptor interactions among basal cells, resident fibroblasts, and immune cells. These signaling pairs drive the coordinated expression of inflammatory mediators and matrix-regulating factors, thereby exacerbating ECM metabolic imbalance within the local microenvironment and accelerating irreversible remodeling processes (70, 71). A recent study integrating macro- and single-cell RNA-seq data further demonstrated that significant expression changes of RNA methylation regulatory factors and autophagy pathways were present in immune cells of COPD patients, and that methylation regulatory factors and autophagy-related genes were upregulated in T cells and macrophages. Furthermore, such epigenetic and autophagy signals were positively correlated with inflammatory factor levels, suggesting that epigenetic modifications and cellular autophagy may serve as upstream regulatory points linking environmental exposure to persistent immune homeostasis imbalance and tissue damage (72).

## Spirometry

In the absence of a non-invasive gold standard for diagnosing small airway obstruction (SAD), traditional pulmonary function tests remain the most commonly used screening tool in clinical practice, mainly because they are easy to operate, reproducible and low-cost (73). In 1972, the maximum mid-expiratory flow (MMEF) was proposed as the best vital capacity parameter for identifying small airway obstruction (SAO) (74). MMEF is commonly referred to as the mean forced expiratory flow between 25% and 75% of forced vital capacity (FEF 25%–75%), which represents the average expiratory flow during this portion of forced vital capacity. It represents the most sensitive measure of airflow in peripheral airways and is indicative of airflow obstruction, which is reduced in the early stages of SAD (75) and it is considered more accurate in detecting small airway disease than the forced expiratory volume in 1 s (FEV1) (76). Its use is grounded in the assumption that the middle and posterior portions of the FVC reflect airflow in small airways, which are susceptible to collapse at the end of expiration due owing to the absence of cartilage support. However, the clinical application of MMEF has remained controversial because of its insufficient sensitivity and specificity for assessing small airways, as well as its dependence on accurate measurement of the FVC (77). To overcome this limitation, some researchers have proposed the ratio of forced expiratory volume in 3 s (FEV3) to forced expiratory volume in 6 s (FEV6) as an alternative indicator for assessing small airway obstruction. The theoretical basis lies in the fact that FEV3 can capture more expiratory volume than FEV1, thereby including gas from the small airways (78, 79). Additionally, the use of FEV6 eliminates the requirement for precise measurement of FVC (80). Currently, the FEV3/FEV6 ratio has been demonstrated to be highly sensitivity for early small airway disease associated with airflow obstruction (3). In the multicenter COPD Genetic Epidemiology (COPD Gene) study, which included former and current smokers, a low FEV3/FEV6 (below the lower limit of normal [LLN]) was linked to the presence of gas trapping on CT imaging and a worse quality of life. This suggests its potential value in clinical screening, especially for early risk identification of former or current smokers. However, it remains uncertain whether these findings translate into increased mortality (81). In addition, studies from large cohorts (e.g., SPIROMICS) have shown that when FEV_3_/FEV_6_ is below the lower limit (LLN), HRCT/PRM-defined gas trapping and functional small airways disease (fSAD) can be detected even in smokers with normal FEV_1_/FVC, suggesting that this simple and easily obtainable respiratory function indicator can help identify potential early small airways lesions (82). Large cohort analyses from the COPDGene cohort also showed that fSAD quantified by CT-PRM was common in subjects who had not yet developed airflow limitation diagnosed by spirometry and was associated with accelerated FEV_1_ decline and adverse clinical outcomes during follow-up, which reinforced the value of imaging fSAD in early identification and risk stratification (83). Therefore, using FEV_3_/FEV_6_ as a preliminary screening in high-risk smoking populations combined with imaging methods such as PRM can help to early identify and stratify the management of individuals with subclinical small airway disease. Owing to its simplicity and low cost, spirometry remains the most commonly utilized diagnostic method for patients with or at risk of developing COPD in clinical management and epidemiological studies (84).

## Single and multiple breath washout experiments

Increased ventilation heterogeneity is a characteristic physiological abnormality observed in respiratory diseases such as asthma and COPD (85, 86). The single-breath washout (SBW) maneuver involves a rapid exhalation to residual volume (RV), followed by inhalation of 100% oxygen to total lung capacity (TLC), and a subsequent exhalation from TLC back to RV, during which inert gas is washed out from the lungs. The most commonly assessed parameter is the slope of phase III (SIII), which reflects the homogeneity of gas clearance across lung regions and helps detect areas of hypoventilation and gas trapping. The operation is relatively simple and can reflect the closure of the small airways (87), but it requires the patient to cooperate with maximal breathing. The results are easily affected by factors such as age and obesity, and the sensitivity is relatively limited. The single-breath washout (SBW) test is suitable for patients capable of performing forced vital capacity maneuvers, such as adults and older children. It requires only one deep inhalation followed by a forced exhalation, making it simple to administer and highly feasible for assessing obstructive diseases such as COPD (88, 89). Studies have shown that the completion rate of SBWZ among children is as high as over 92%, which is significantly faster than MBW (83%) (89). Its limitations include the need for a high level of patient cooperation, low sensitivity in detecting mild small airway lesions, and the strong dependence of single washout parameters (such as SIII) on the subject’s effort. Multiple-breath washout (MBW) evaluates pulmonary gas mixing and clearance over repeated inspiratory and expiratory cycles, offering a comprehensive assessment of ventilation heterogeneity. Because it requires only quiet tidal breathing–without breath-holding or forced exhalation–it is particularly well suited for young children, infants, and patients unable to perform spirometry (90). Key parameters include the lung clearance index (LCI), Scond, Sacin, and functional residual capacity (FRC), which respectively reflect overall ventilation heterogeneity, convection-dependent heterogeneity in the conducting airways, diffusion-dependent heterogeneity in the peripheral airways and alveolar regions, and theresting end-expiratory lung volume (91). Over the past two decades, a large body of literature has emerged on the LCI, demonstrating that it is more sensitive than spirometry in detecting early obstructive lung disease in children with cystic fibrosis (CF) (92–94). MBW testing is time-consuming (average 2–5 min per test for healthy subjects (90), typically requires three or more repeats to ensure data reliability (90), and requires specialized gas analysis equipment and strict quality management. Nevertheless, MBW is widely applied in children and in the screening of early-stage conditions such as cystic fibrosis and primary ciliary dyskinesia, as it requires minimal patient coordination and is highly sensitive to subclinical small airway disease (95–97). Clinically, the two can be combined to evaluate small airway lesions, or combined with other examinations for a more accurate diagnosis.

## Impulse oscillometry system

Forced oscillations are pressure or flow signals, or waves, that originate below the human hearing range, typically spanning 5–37 Hz, and are superimposed on normal tidal breathing, providing information about resistance and reactance on the basis of respiratory mechanics, i.e., airflow obstructions both inside and outside the bronchi. The use of multiple wave frequencies enables the exploration of the impedance frequency dependence to more precisely describe the location of airway obstruction. However, recent studies have shown that the impedance frequency dependence increases with disease severity or is influenced by the patient’s chest wall stiffness (87). This method tends to underestimate the true condition of lung mechanics (98–103). Currently, the latest pulse oscillation method is employed to assess small airway size by analyzing the heterogeneity of low-frequency resistance at 5–20 Hz (R5-R20), low-frequency reactance at 5 Hz (X5), or the area under the reactance curve between 5 Hz and the resonant frequency (100). This is because, at high oscillation frequencies (20 Hz), the signal is dominated by the characteristics of the upper airways, whereas at low frequencies (below 10 Hz), the signal reflects the entire tracheobronchial tree, including small airway resistance. At lower oscillation frequencies, reactance reflects the elastic properties of the parenchyma, airways, and chest wall, whereas at higher frequencies, inertial forces become predominant. At the resonant frequency (8 Hz), the elastic and inertial forces are equal and opposite, at which point they cancel each other out, and the pressure-flow relationship at this frequency reflects only the resistance of the system (104). Compared with spirometry, this method does not require much effort from the patient. It can also accurately detect small airway disease in all age groups (3, 105). In addition, impulse oscillometry has been shown to be more sensitive than spirometry in detecting small airway disease. Spirometry and impulse oscillomerty are recommended in combination for hospitalized patients to assess small airway function comprehensively and enable timely intervention (106). The use of an impulse oscillome try system has been more extensively studied in obstructive airway diseases. Recent studies have demonstrated that impulse oscillometry can be utilized to detect early manifestations of chronic obstructive pulmonary disease (COPD) (107). It is more useful than emphysema in the assessment of disease and can identify small airway dysfunction in patients with normal pulmonary function but early symptoms of chronic obstructive pulmonary disease (COPD) (76, 108). Moreover, the incidence of respiratory symptoms has been shown to be significantly greater in patients with small airway disease (SAD), as defined by impulse oscillometry (76). Correlations between impulse oscillometry (IOS) parameters and clinical indicators, including the severity of bronchiectasis and the presence of potential pathogenic microorganisms in sputum (109, 110). Park demonstrated that impulse oscillometry (IOS) may be a more sensitive method for predicting disease progression and prognosis than traditional lung function tests are. It can also differentiate between small airway disease (SAD) and chronic obstructive pulmonary disease (COPD), serving as an effective tool for assessing the risk of acute exacerbations in both asthma and COPD patients. In the clinical management of elderly patients with chronic obstructive pulmonary disease (COPD), impulse oscillometry (IOS) offers several advantages (111). A prospective study demonstrated that patients with small airway disease (SAD) defined by impulse oscillometry (IOS) experienced a more rapid decline in lung function and a greater risk of acute exacerbations (112). However, the consistency of results from oscillometric and other small airway detection methods in diagnosing small airway dysfunction remains a topic of ongoing discussion.

## Frequency dependence of dynamic lung compliance

Dynamic lung compliance (Cdyn) refers to the ratio of changes in lung volume to the corresponding changes in airway pressure during respiration. Unlike static compliance, which purely reflects the elasticity of lung tissue and is not affected by airway resistance, dynamic compliance reflects the combined effects of lung tissue elastic resistance and airway resistance. In clinical practice, pulmonary function tests are frequently used to measure end-expiratory and end-inspiratory lung volumes and pressures in real time. Dynamic compliance (Cdyn) is calculated by dividing tidal volume by the difference between peak inspiratory pressure and end-expiratory pressure (113). Dynamic compliance includes the resistance to airflow through the airways in addition to the elasticity of lung tissue and is therefore usually lower than static compliance (114). Dynamic compliance reflects the combined influence of lung tissue elasticity and airway resistance and is commonly used in clinical practice to evaluate their interactive effects. Under normal conditions, the time constants of each alveolar unit are closely aligned, causing dynamic compliance to remain nearly constant as the respiratory rate increases from a resting state (≈8–16 breaths/minute) to an active state (≈60–120 breaths/minute). In other words, the normal lung time constant (τ = R⋅C) is both uniform and brief, ensuring synchronized inflation and exhalation of each lung area and thereby maintaining a constant Cdyn value regardless of changes in respiratory rate. This phenomenon demonstrates that, in healthy individuals, the respiratory rate and dynamic compliance are largely independent (104). When small airway obstruction or dysfunction develops, this uniformity is disrupted, resulting in the progressive appearance of frequency-dependent dynamic compliance (115). An early study by Woolcock (116) et al. found that even if the routine lung function indicators of patients with chronic bronchitis or asthma were close to normal, their dynamic compliance was significantly reduced at higher respiratory rates, while there was no such change in the healthy control group. These results suggest that airway obstruction leads to uneven ventilation, preventing some lung regions from being adequately deflated during rapid breathing, thereby reducing overall compliance. Thus, in patients with small airways disease, dynamic compliance typically decreases with increasing respiratory rate, disrupting the normal rate independence. The frequency dependence of dynamic compliance provides a sensitive indicator for the early diagnosis of small airway disease. As early as 1969, Woolcock et al. discovered latent airway obstruction through compliance testing at different respiratory rates (116). In recent years, with growing attention to COPD and early small airway disease, researchers have sought to integrate traditional compliance measurements with novel respiratory impedance techniques, such as oscillometry. The oscillometric low-frequency reactivity (AX) is closely related to the elasticity of peripheral lung units and is considered to reflect small airway patency and lung compliance (108). Teixeira et al. (117) used oscillometry to measure the Cdyn of patients with both chronic bronchitis and COPD, which was significantly lower than that of the healthy control group. This indicates that the decrease in dynamic compliance is related to the severity of airflow limitation. In the future, we can try to integrate static compliance and oscillometry to further improve the sensitivity of early detection of small airway disease and provide more basis for early intervention of COPD.

## HRCT/CT and PRM

High-resolution computed tomography (HRCT) is the most effective imaging modality for assessing small airway disease (Figure 1). Direct signs of small airway disease observed on high-resolution computed tomography (HRCT) scans result from alterations in the airway wall or lumen. On HRCT scans, abnormal small airways may present as tubular, nodular, or branching linear structures. Indirect signs of small airway disease result from alterations in the lung parenchyma distal to the affected small airways and include air trapping, subsegmental atelectasis, centrilobular emphysema, and nodules (118). However, the spatial resolution of HRCT is limited (approximately 2 mm), making it generally impossible to directly visualize the smaller terminal bronchioles. As a result, early small airway lesions are often difficult to detect on conventional HRCT images. In contrast, parametric response mapping (PRM) is a quantitative imaging technique based on the registration of paired inspiratory and expiratory scans (119). This method classifies each registered lung voxel according to the HU value of the inspiratory and expiratory CT: it not only identifies the low-density area caused by emphysema, but also marks the emphysematous gas retention area of the lung (functional small airway disease, fSAD), and generates the corresponding 3D map (120). Compared with traditional spirometry, PRM has the advantages of spatial visualization and numerical reproducibility. Many studies have used the volume percentage of fSAD in PRM representing non-emphysematous gas retention (PRM^fSAD^) as an indicator for evaluating small airways (83, 121–124), including analyses from large cohorts such as COPDGene. Small airway disease is a transitional stage in the progression from normal airways to emphysema in severe disease, as demonstrated by the follow-up Parametric Response Mapping (PRM) study in the SPIROMICS cohort, which used inspiratory and expiratory HRCT scans to assess small airway disease (120, 125). A previous study compared 78 COPD patients at different stages of SAD and demonstrated that small airway stenosis and obstruction appear earlier than emphysema, so HRCT has advantages in the early diagnosis of COPD (126). The advantage of PRM is that it provides spatially resolved functional imaging and reproducible quantitative indicators, which go beyond the capabilities of traditional lung function tests (which can only give average results of the whole lung) (120, 127). PRM analysis can clearly visualize the spatial distribution of gas trapping and differentiate the causes of gas trapping (small airway disease or emphysema), facilitating the differential diagnosis of different COPD phenotypes (120). Compared with simple lung function measurement, PRM also has better reproducibility and objectivity (127). Therefore, in smokers or other high-risk groups, when screening, grading, or follow-up of early small airway lesions is required, PRM can be considered to obtain more functional information than conventional HRCT. However, PRM also has limitations. First, it requires two scans, one for inspiration and one for expiration, which increases examination time, cost, and radiation exposure (119), and therefore has not yet become a routine examination method. Secondly, PRM results rely on accurate image registration, and its measurements are based on changes in lung parenchymal density, which may be interfered with by changes in pulmonary vascular or other tissue density, affecting the relevance of small airway disease detection (127). Furthermore, PRM reflects functional gas trapping rather than direct structural imaging, so anatomical details such as bronchial wall thickening and mucus plugging require HRCT assessment. Overall, HRCT and PRM are complementary examination methods, capable of visualizing changes in lung structure and assessing small airway dysfunction Therefore, combining patient information and HRCT to assess SAD still has good predictive value. At the same time, some studies have found that PRM^fSAD^ may indicate early alveolar attachment loss. Although previous studies support the relationship between PRM and alveolar attachment (121), this deserves further exploration. Currently, recent studies have shown that generative AI techniques can be used to reliably assess small airway disease using deep learning in inspiratory chest CT, without the need for additional respiratory CT scans. The fSAD obtained from inspiratory CT is strongly correlated with PRM^fSAD^ and is significantly associated with a decrease in FEV1, while providing higher reproducibility (128).

## SPECT

Quantitative single-photon emission computed tomography (SPECT/CT) can objectively quantify regional heterogeneity in human ventilation and is more sensitive than chest CT and lung function tests in detecting early airway changes in COPD patients. The coefficient of variation (CV) of the distribution of radioactive tracer values inhaled during the test can generate heterogeneity maps and density curves for small lung regions (129, 130). The area under the coefficient of variation (CV) curve (AUC-CV) at a predetermined threshold serves as a marker of ventilation in homogeneity and may have clinical value in assessing the severity and distribution of lung disease. Studies have shown that AUC-CV values are sensitive to the presence of COPD, asthma, and airflow obstruction, and are correlated with even mild abnormalities in pulmonary function tests (PFTs), including in otherwise healthy individuals. Therefore, measuring the AUC-CV value may serve as an early marker of airway disease in active smokers at risk for COPD (131, 132). A study by Juneau demonstrated that the AUC-CV40% can serve not only as a marker for peripheral airway disease but also as a clinical indicator for respiratory symptoms. It can also serve as an auxiliary tool for clinicians to assess smokers with respiratory symptoms. However, whether this marker can serve as a prognostic indicator for COPD requires further investigation (133). In fact, although Technegas technology has benefited more than 4.4 million patients in more than 60 countries since it was first used in Australia in the 1980s, it has only recently been approved for use in some regions (such as the United States) (134). In addition, current guidelines mainly recommend SPECT/CT for evaluation of pulmonary embolism rather than COPD screening (134). Current guidelines mainly recommend SPECT/CT for the evaluation of pulmonary embolism, rather than COPD screening (135). Therefore, SPECT ventilation imaging is currently mostly used in special occasions such as scientific research and preoperative evaluation of pulmonary surgery, and has not yet been included in the routine diagnostic process of COPD (136, 137). In summary, although SPECT/CT ventilation imaging can help detect airway function changes early, it is still mainly a research tool due to the high complexity of its equipment and operation and strict requirements for tracers, and its clinical practicality and maturity are limited.

## Perfluoropropane-based ^19^F MRI and ventilation defect percentage (VDP)

Perfluoropropane 19F MRI is emerging lung ventilation imaging technique that uses inert fluorinated gases, which are detectable in MRI (Figure 1), to depict lung ventilation distribution. After subjects inhale a gas mixture containing approximately 79% perfluoropropane and undergo MRI scanning, the spatial distribution of the 19F signal during lung inflation is obtained and quantified as the ventilation defect percentage (VDP). Pippard and Neal developed a novel technique to assess the ventilation defect percentage (VDP), which reflects air trapping in the small airways. The subjects inhaled perfluoropropane gas and underwent fluorine-19 (^19^F) MRI scans. Inspiratory fluorine-19 (^19^F) MR images revealed regions of poor lung ventilation, as indicated by the heterogeneity in the distribution of perfluoropropane. VDP was calculated by dividing the volume of aerated lungs displayed on the inspiratory fluorine-19 (^19^F) MRI scan by the volume determined on the proton MRI scan (excluding the trachea and large airways). In their study, after bronchodilator inhalation, the VDP of asthma patients decreased by an average of 33%, whereas that of COPD patients decreased by an average of 14%. The FEV and FVC reflect airway disease, whereas VDP provides insights into regional air trapping and dead space, particularly in the small airways. Unlike FEV1 and FVC, which mainly reflect global airflow limitation, VDP can reflect local gas trapping and dead space, and is more sensitive in small airway lesions. Compared with other detection methods, such as hyperpolarized MRI or helium (He) MRI, perfluoropropane ^19^F MRI technology is straightforward, cost-effective, and capable of accurately depicting the distribution of lung gas during both inhalation and exhalation. It also demonstrates good tolerance in test subjects and maintains high clinical safety. Furthermore, perfluoropropane is an inert gas that is not metabolized and is rapidly cleared through exhalation, making this method safer for lung imaging and ensuring that it does not cause lasting effects on the body. Overall, this is a promising, investigational approach that can sensitively detect regional ventilation defects, but its availability is currently limited and has not yet been widely used in routine clinical practice. This imaging technology aids in detecting the early stages of COPD and asthma and may facilitate early intervention to enhance outcomes and more effectively evaluate treatment effects (138, 139). A review pointed out that although lung ventilation imaging is the most common case of 19F MRI in clinical practice, it is still relatively rare overall. In addition, the operation requires the subject to hold their breath for a short time to obtain clear images, which poses a tolerance challenge for patients with severe COPD. To this end, the research team is developing fast imaging and reconstruction algorithms to shorten scanning time and reduce the need for breath holding (138).

Although conventional spirometry is widely used and cost-effective, its sensitivity for detecting early small airway disease (SAD) remains limited. Many patients with pathological changes may still present with preserved FEV1 and FVC values, thereby delaying the recognition of early or pre-COPD phenotypes. Emerging techniques such as multiple-breath washout (MBW) and impulse oscillometry (IOS) allow non-invasive assessment of ventilation heterogeneity and peripheral airway resistance, offering superior sensitivity for the early detection of SAD. Imaging-based approaches, including parametric response mapping (PRM), single-photon emission computed tomography (SPECT), and ^19^F magnetic resonance imaging (^19^F-MRI), provide high-resolution insights into regional small airway obstruction and remodeling. However, their clinical application remains constrained by high costs, limited availability, and the need for further validation. Overall, these methods complement spirometry by bridging the diagnostic gap between pre-COPD and early COPD, thereby facilitating earlier intervention strategies and potentially improving patient outcomes (Table 1).

**TABLE 1:** Comparison of different pairs of assays.

Modality	Sensitivity*	Availability	Radiation	Relative cost	Stage of validation
Spirometry (including FEV_1_/FVC;FEV_3_/FEV_6_)	Low (often normal in early disease)	Widely available	None	Low	Established; limited sensitivity for early detection
MBW	Moderate–High	Limited (specialized/research centers)	None	Moderate	Increasing validation; mainly research use
IOS	Moderate	Moderate availability (selected centers)	None	Moderate	Growing clinical adoption, esp. pediatrics and COPD research
HRCT/CT-PRM	High (sensitive to fSAD)	Requires advanced CT capability and post-processing	Yes ionizing radiation; PRM typically requires paired inspiratory + expiratory CT (two scans)	High	Under validation; promising for pre-COPD phenotyping
SPECT	High	Limited (nuclear medicine centers)	Yes radiotracer exposure	High	Mainly research use; not routine clinical practice
^19^F-MRI	Very High (highly sensitive to regional ventilation defects)	Very limited (research institutions)	None	High	Early validation; investigational and promising; requires breath-hold/technical optimization

Sensitivity*: refers to early SAD/pre-COPD (often with normal FEV_1_/FVC).

## Therapeutic perspectives

Chronic airway inflammation represents the core pathological basis and primary driving factor in the progression of chronic obstructive pulmonary disease (COPD) and various small airway diseases. It is characterized by inflammatory cell infiltration and excessive production of proinflammatory cytokines, which contribute to airway remodeling, airflow limitation, and acute exacerbations of the disease (140, 141). Given this underlying mechanism, anti-inflammatory therapy has emerged as a central strategy in the management of these conditions, aiming to reduce inflammation, alleviate symptoms, and slow disease progression (140, 141). Anti-inflammatory drugs primarily function by targeting key inflammatory signaling pathways–such as nuclear factor-κB (NF-κB) and mitogen-activated protein kinase (MAPK)–modulating the balance between proinflammatory and anti-inflammatory mediators, inhibiting the activation and migration of inflammatory cells, and ameliorating oxidative stress and protease/antiprotease imbalance (141–143). These agents can block the activation of NF-κB and MAPK pathways, thereby reducing the transcription and release of proinflammatory cytokines including interleukin-6 (IL-6) and tumor necrosis factor-α (TNF-α). Additionally, they can modulate the redox state via histone deacetylase 1 (HDAC1)-dependent mechanisms, thereby attenuating oxidative damage in lung tissue (141, 142, 144). Inhaled glucocorticoids (ICS) are a cornerstone of anti-inflammatory treatment in COPD. They exert their effects by binding to the glucocorticoid receptor (GR), inhibiting the transcription of proinflammatory genes (e.g., TNF-α, IL-6), and activating anti-inflammatory genes. COPD patients with elevated blood eosinophil counts (BECs) show a better response to ICS treatment, an effect associated with the suppression of type 2 inflammatory pathways rather than direct reduction in eosinophil numbers (145). Antifibrotic agents constitute a class of compounds that interfere with the fibrotic process. Their mechanisms include inhibiting the activation and proliferation of fibroblasts and myofibroblasts, regulating extracellular matrix (ECM) synthesis, and suppressing profibrotic inflammatory responses. Specific actions involve blocking signaling pathways such as transforming growth factor-β (TGF-β) and cyclic adenosine monophosphate/protein kinase A (cAMP/PKA), reducing ECM synthesis by inhibiting synthases, and modulating inflammatory mediator secretion to alleviate inflammatory responses (146, 147). Studies indicate that targeting the TGF-β pathway can inhibit fibroblast-to-myofibroblast differentiation and reduce ECM deposition (148). For instance, pirfenidone inhibits TGF-β-induced Smad3, p38, and protein kinase B (Akt) phosphorylation, thereby suppressing fibroblast proliferation and collagen production (146). Epigallocatechin gallate (EGCG), a green tea polyphenol, demonstrates antifibrotic potential in respiratory diseases by inhibiting the TGF-β1 pathway, downregulating α-smooth muscle actin (α-SMA) and collagen expression, and reducing ECM deposition. In a bleomycin-induced pulmonary fibrosis model, EGCG reduced hydroxyproline content and enhanced antioxidant enzyme activity, mitigating pulmonary fibrosis (149). BI 1015550, a phosphodiesterase 4B (PDE4B) inhibitor, exerts antifibrotic effects by inhibiting TGF-β-stimulated myofibroblast transformation and ECM protein expression in fibroblasts from idiopathic pulmonary fibrosis (IPF) patients (150). Nintedanib, a tyrosine kinase inhibitor, inhibits fibroblast proliferation and exhibits synergistic effects with BI 1015550 (150, 151). Moreover, nintedanib inhibits collagen fiber assembly, representing a novel antifibrotic mechanism (151). Senolytics are small molecules or biological agents that selectively induce apoptosis in senescent cells (SCs) without affecting non-senescent cells (152). These drugs were initially identified via a “network targeting” strategy: senescent cells accumulate with age or disease and upregulate anti-apoptotic pathways (SCAPs) such as B-cell lymphoma-2/extra large (BCL-2/BCL-XL), phosphatidylinositol 3-kinase/protein kinase B (PI3K/AKT), hypoxia-inducible factor-1α (HIF-1α), and receptor/tyrosine kinase-dependent pathways, counteracting the pro-apoptotic effects of the senescence-associated secretory phenotype (SASP) (152, 153). Senolytics eliminate senescent cells by transiently inhibiting SCAPs, inducing mitochondrial outer membrane permeabilization and caspase-mediated cell death, while sparing healthy cells (153). In a bleomycin-induced mouse model of pulmonary fibrosis (simulating IPF), the combination of dasatinib and quercetin (D + Q) reduced senescent fibroblasts and epithelial cells, decreased expression of fibrotic factors such as type I collagen α1 chain (Col1a1) and TGF-β, and improved lung compliance (154). Multiple *in vivo* and *in vitro* studies have shown that senolytics (such as D + Q) can reduce the burden of senescent cells in the respiratory epithelium or mouse models and improve inflammatory indicators, but efficacy and safety data at the population level are still limited and no consensus has been reached. Some small sample or early trials have shown positive signals, but data from larger-scale, randomized controlled trials are still scarce, so its clinical feasibility and long-term risks need to be carefully evaluated (155, 156). In a cigarette smoke-induced lung injury model using p16-3MR mice, ganciclovir (GCV) eliminated p16^+^ senescent cells, inhibited neutrophil inflammation mediated by the C-X-C chemokine ligand 1-keratinocyte chemoattractant (CXCL1-KC) axis, and restored mitochondrial function. This intervention reduced senescence-associated β-galactosidase-positive (SA-β-Gal^+^) cells, reversed lung aging, diminished neutrophil infiltration in bronchoalveolar lavage fluid, and ultimately attenuated smoke-induced alveolar enlargement, highlighting its potential value in early COPD intervention (157).

## Conclusion

Small airway disease (SAD) is present throughout the course of COPD but plays a particularly critical role in its early development. Three interrelated pathological mechanisms drive injury and remodeling of the small airways. First, chronic inflammation–primarily mediated by neutrophils and macrophages–directly damages the epithelium and extracellular matrix (ECM) through proteases (e.g., MMPs, elastase), chemokines, and reactive oxygen species, thereby promoting structural destruction and airway narrowing. Second, fibrosis and epithelial–mesenchymal transition (EMT), activated by profibrotic pathways such as TGF-β, induce fibroblast-to-myofibroblast transdifferentiation and excessive ECM deposition. Third, the senescence–SASP axis, whereby senescent cells amplify local inflammation and propagate paracrine senescence through the secretion of SASP factors (e.g., IL-6, IL-8, PAI-1, MMPs), synergistically accelerates remodeling and functional decline. These mechanisms do not act in isolation but continuously interact within the small airway microenvironment, collectively driving the progression from SAD to overt COPD (55, 158, 159).

To achieve “early detection, early diagnosis, and early treatment,” the combination of HRCT and PRM has been shown to differentiate functional small airway disease (fSAD) from emphysema at the voxel level and predict subsequent functional and structural progression. The latest deep learning methods can even approximate PRM information using only inspiratory CT, significantly reducing the need for additional respiratory scans (119, 125). MBW and IOS are more sensitive for detecting airway malfunction and peripheral airway resistance when spirometry is normal (97, 160), and can be considered for use in outpatient clinics or longitudinal follow-up. Furthermore, single-cell omics has revealed the spatiotemporal landscape of small airway cell subsets and immune-stromal interactions, laying the foundation for linking imaging phenotypes with molecular mechanisms. These complementary approaches enhance the sensitivity of small airway detection.

Future therapeutic strategies for COPD should focus on disrupting key pathogenic circuits of small airway disease. Promising approaches include: senolytics or senomorphics to attenuate SASP-driven inflammation and paracrine senescence; microRNA- or exosome-based precision interventions to restore anti-senescence pathways; antifibrotic, anti-TGF-β, and antioxidant therapies to limit ECM deposition and oxidative damage; and targeting immune regulatory axes, such as neutrophil chemotaxis/activation or PI3Kδ signaling, to reduce protease-mediated injury (55, 156, 159, 161).
