# Supplementary figures and images for: Small airway disease as a key factor in COPD: new perspectives and insights
Source: Front Med (Lausanne). 2025 Sep 26;12:1648612. doi: 10.3389/fmed.2025.1648612 (PMC12510933; doi:10.3389/fmed.2025.1648612)

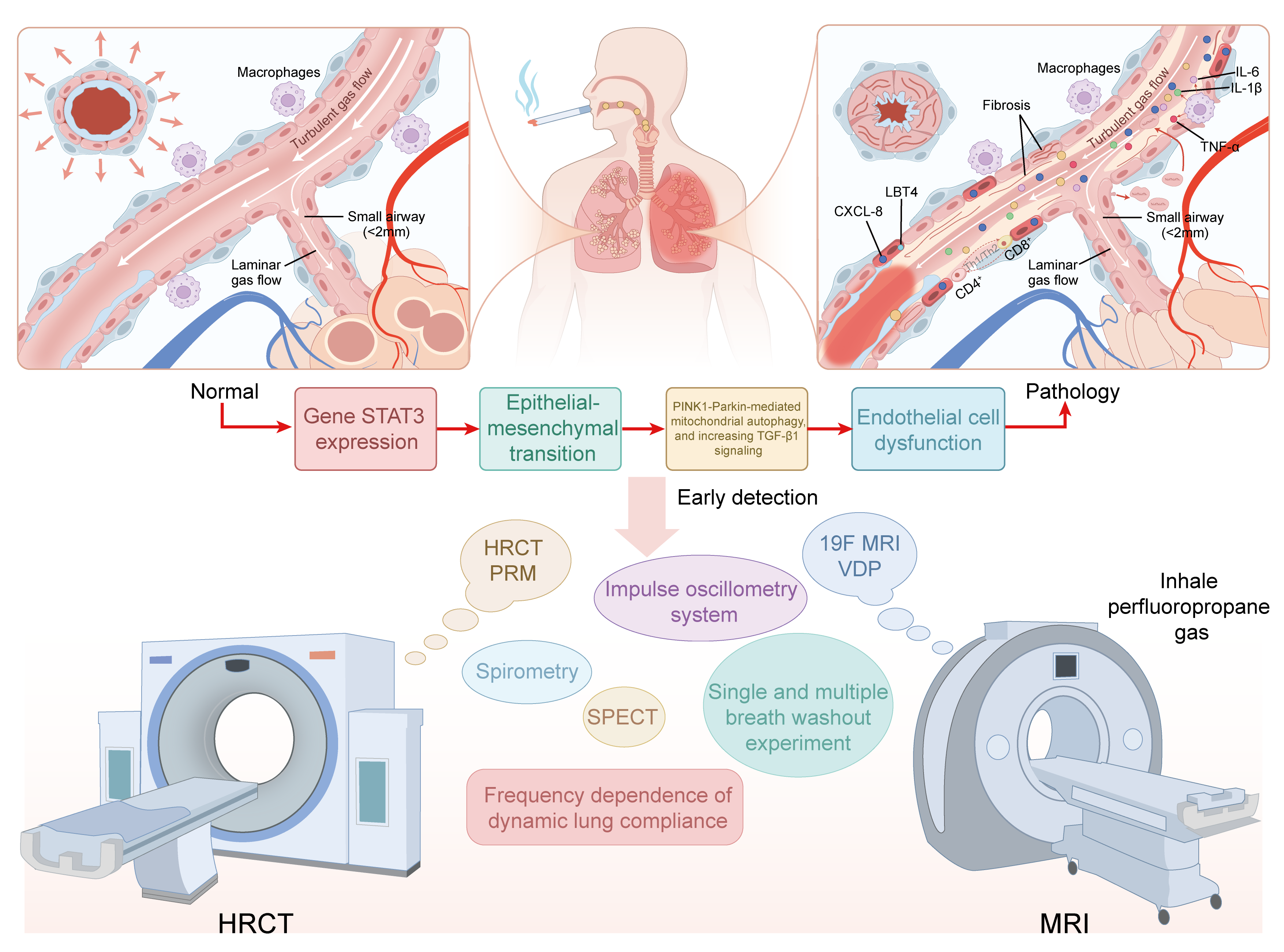

Supplement: Supplementary file 1 [file Image_1.png]
